# Feasibility, acceptability, patient experience, and preliminary efficacy of a virtual reality guided imagery intervention for chronic pain

**DOI:** 10.3389/fdgth.2025.1505861

**Published:** 2025-06-24

**Authors:** Lauren Doan, Marc Recasens, Jessica Lake, Ian Miller, Elise Vierra, Steven Richeimer, Iris Yao, Doerte U. Junghaenel, Faye Weinstein

**Affiliations:** ^1^ Department of Science, Limbix Health Inc., San Francisco, CA, United States; ^2^ Department of Research, BehaVR Inc. (DBA RealizedCare), Elizabethtown, KY, United States; ^3^ Department of Content Design, Limbix Health Inc., San Francisco, CA, United States; ^4^ Department of Anesthesiology, University of Southern California, Los Angeles, CA, United States; ^5^ Department of Psychology and Dornsife Center for Self-Report Science, University of Southern California, Los Angeles, CA, United States; ^6^ Department of Psychiatry, University of Southern California, Los Angeles, CA, United States

**Keywords:** virtual reality, guided imagery, chronic pain, complex regional pain syndrome, chronic back pain

## Abstract

**Objectives:**

Guided imagery is a strategy utilized in chronic pain management by patients. Benefits are cumulative via ongoing application. Engagement via Virtual Reality (VR) is becoming more accessible as a strategy to enhance adherence, use and benefit of guided imagery. We conducted a preliminary investigation of the feasibility, acceptability, patient experience, and efficacy of the use of VR for patients with chronic pain to use at home.

**Methods:**

36 patients with Complex Regional Pain Syndrome or Low Back Pain were randomly assigned to VR or audio only guided imagery groups. Feasibility, acceptability and patient experience were rated by participants. Outcomes assessed at baseline and post-intervention were pain, mental and physical health, and mood.

**Results:**

Results indicate that the intervention was feasible and found acceptable by participants. The intervention also demonstrated promising preliminary efficacy based on self-reported within-group decreases in pain, depressive symptoms, anxiety symptoms and improvements in physical and mental functioning.

**Conclusions:**

The use of VR shows promise for enhancing the application and experience of guided imagery training with people who have chronic pain.

## Introduction

Addressing chronic pain with behavioral techniques and self-regulation strategies is crucial to promote greater outcomes in multidisciplinary pain management. Self-regulation strategies for chronic pain, including guided imagery, require learning, skill building, and independent application by the patient. Self-directed guided imagery (GI) is a cognitive behavioral technique where individuals are instructed through guided narration to create sensory-rich images (imaging ability) in their mind to alter their psychological and physiological states to reduce their experience of pain. Instruction for guided imagery typically includes specific directions for strategies aimed at reducing autonomic arousal and shifting to positive cognitions (1) and is typically delivered via audio instruction or by a person reading a script to themselves. Repeated efforts by the patient over multiple sessions are often needed to engage mechanisms, like self-efficacy, mastery, and self-regulation, to achieve meaningful outcomes in managing chronic pain (2, 3). Research has shown a cumulative effect of GI on health outcomes, including pain, mood, and quality of life, with repeated and frequent practice sessions (4, 5).

Despite these advantages to using GI for managing chronic pain, limitations in present GI approaches exist. Critically, GI depends on the ability of patients to imagine vivid experiences in their mind. Individual differences in imaging abilities can therefore affect the success of GI in reducing pain (6–10), increasing variability in treatment response and reducing the potency of this intervention for many patients.

Research evidence suggests that virtual reality (VR) is safe (11) and effective for managing acute pain and chronic pain (12–16), including for severe levels of pain (17). A recent scoping review by Ding et al. (18) maps the breadth of literature on virtual reality and chronic pain, identifying existing research on pain types, VR interventions, immersion and side effects for 36 studies that met criteria.

Theoretical perspectives suggest that VR reduces pain via top-down modulation of pain pathways by cognitive and emotional brain regions (19). Pain perception requires attention, which is a limited cognitive resource, thus diversion of attention from pain is believed to mechanistically account for how distraction reduces pain (20). Evidence that active VR experiences more effectively reduce pain than passive VR experiences (21) supports this idea, as active VR experiences engage more attentional resources than passive ones. Immersive, multisensory VR experiences that heighten the sense of presence in a virtual environment also engage emotional regions of the brain. Given evidence that positive emotions decrease pain, (22) the influence of emotional regions on pain pathways may be a second mechanism by which VR experiences modulate pain. Preliminary neuroimaging evidence supports modulation of pain-related brain activity by VR experiences (23).

As a delivery method for GI, VR may be particularly useful not just for distraction, but also due to the immersive qualities of the technology. VR currently requires the use of a head mounted display, which facilitates a strong sense of immersion with a highly stimulating visual experience. Immersion describes a feeling of engagement and presence by the user in the VR activity, visual and/or auditory feedback, and emotional investment in the form of challenges and interactivity (24, 25). VR users also benefit from positive affective components and the narrative-sequential immersion, which encourages users to continue to engage in the activity to see how events progress. This facilitation in experiential practice efforts and engagement by the VR user increases the potential to enhance learning and improve independent application of GI (26).

With some exceptions, research on VR interventions has been conducted in clinic or laboratory settings, requiring valuable staff or clinician time (27). Studies assessing the feasibility of VR interventions in clinical settings demonstrate a positive perceived effectiveness, usability, acceptability, and endorsement of VR interventions in patients with chronic pain, suggesting that VR may be an enjoyable alternative to traditional physiotherapy (28–31). However, some studies have reported poor recruitment and high dropout rates in clinical settings (30, 31), highlighting barriers in VR adoption by healthcare providers, such as lack of reimbursement or challenges integrating VR into their clinical workflows (32, 33).

Advances in VR technology have enabled high quality and affordable consumer VR headsets. At-home treatments may therefore provide a viable pathway of delivery, although existing research is limited. A retrospective analysis of the impact of at-home VR intervention on patients with neck and back pain found improvements in pain intensity, anxiety and depression. However, the study did not include a control condition and the VR intervention was guided by a behavioral health clinician (34). In a randomized controlled trial comparing a skills-based VR program to a VR sham control in patients with chronic low back pain researchers found less than 10% dizziness in both groups, significant improvements in pain intensity, sleep, pain interference, and increased satisfaction in the active group (35). Though well-controlled, this study doesn't allow for an evaluation of the additive value of VR relative to more traditional guided imagery.

To our knowledge, only two studies have compared VR guided imagery to an audio only control condition. Feasibility and preliminary efficacy of at-home, repeated use VR interventions utilizing pain management education, cognitive behavioral therapy (CBT) and self-regulation training skills specific to chronic pain was examined by Darnall et al. (36) who found high engagement and satisfaction ratings coupled with a 30% reduction in pain intensity in a 21-day trial. Using a similar protocol in patients with sickle cell disease, Matthie and colleagues (37) reported high engagement and acceptance ratings, with minor symptoms of cybersickness. Collectively, these studies suggest the potential for VR to facilitate pain relief through skills mastery via at-home repeated sessions, though further evaluation of feasibility, acceptability and patient experience is warranted and other pain populations should be evaluated to understand the generalizability of these findings.

To address these gaps, the current study was designed to explore the feasibility, acceptability, patient experience, and preliminary efficacy of using at-home VR-GI vs. a matched auditory only guided imagery control in patients with chronic back pain and complex regional pain syndrome. This design allowed for an evaluation of the potential additive effect of VR on outcomes. This study addresses the issue of limited research on the use of skills-based VR that employs principles of guided imagery, self-regulation training and CBT via content that was specifically developed to address all of these areas. Due to the high prevalence of effective VR interventions in numerous medical applications and preliminary evidence of acceptability in in-home settings (36), it was anticipated that the VR-GI intervention would be feasible and acceptable for patients with chronic pain.

## Materials and methods

### Recruitment

36 participants [26 female, 10 male; mean age (SD) = 54.9 (12.8) years] were recruited from the pain center at a large, urban academic medical center by physician referral. Guidelines suggest that 12 participants per group is sufficient for pilot studies, as precision gains for point and variability estimates largely stabilize around this value (35). Thus, a total sample size of 12 was targeted for the control condition and a sample size of 24 was targeted for the experimental arm to ensure a minimum of 12 participants with chronic back pain (CBP) and 12 participants with complex regional pain syndrome (CRPS) were randomized to the VRGI group to ensure that feasibility could be evaluated within each group. Criteria for participant inclusion were (1) English fluency, (2) diagnosis of chronic back pain (CBP) or complex regional pain syndrome (CRPS), (3) average pain intensity of 5 or greater on a 0–10 scale for more than 3 months, and (4) access to a device with video and audio capability along with sufficient Wi-Fi. Criteria for participant exclusion were, per participant's self-report, (1) children and adolescents under the age of 18, (2) history of significant motion sickness, (3) active nausea/vomiting, (4) epilepsy, (5) significant movement problems, and (6) significant vision or hearing impairment.

Interested participants were scheduled for a virtual pre-screening on a phone or video call to assess initial eligibility. Eligible participants then scheduled an appointment through an online video platform (Zoom) where the procedures, requirements, benefits and risks associated with the study were described. Participants provided electronic signed informed consent if they agreed to participate.

### Intervention settings and study procedure

After meeting eligibility criteria and providing informed consent, participants were randomized within diagnosis to receive either VR-GI or audio-only GI. Enrollment goal was for 24 participants in the VR-GI group and 12 participants in the audio only group. An additional enrollment goal was to have equal numbers of participants with back pain and CRPS in each group. Participants in the VR-GI group were mailed a VR headset preloaded with the VR-GI program, a tablet, and earbuds. Participants in the audio-only group (AO-GI) were mailed a tablet preloaded with a playlist with GI audio tracks and earbuds.

In a virtual onboarding session with one of the members of the research team, participants completed online baseline assessments of pain intensity, functional outcomes, quality of life, and mood through a REDCap based survey (38). Additionally, participants received basic training in how to complete VR-GI or AO-GI sessions at home using MyCap, a REDCap participant-facing mobile application used to collect patient-reported outcomes (38). All participants were instructed to practice GI at least once per day for 2 weeks. Participants in both groups were instructed to sit in a comfortable position, ideally in a swivel chair for the VR group; standing was discouraged. During onboarding, the “Garden” experience was completed with the research associate and the participant was instructed to complete the “Choose Your Own Adventure” experience the next day. After the first two days, participants were then allowed to complete either experience for the remainder of the 2-week intervention study period. After 2 weeks, participants returned their equipment and completed online post-intervention assessments of pain intensity, pain medication use, functional outcomes, and mood. Those in the VR group were then asked to complete a self-report questionnaire that included questions regarding the comfort, ease of use, overall recommendations, and any adverse effects of the intervention.

Participants were compensated for their participation with Amazon gift cards. Participants were given $10 for attending the onboarding session, $10 for completing the follow-up appointment and $50 for returning the study equipment. Participants could earn a total of $70 for completing all appointments and returning the equipment.

All study procedures were reviewed and approved by the Institutional Review Board (IRB) of the University of Southern California (Study ID: HS 1900549). The study was registered with ClinicalTrials.gov: NCT04849897 Virtual Reality Guided Imagery for Chronic Pain (VRGI).

#### Equipment

The VR headsets used were PICO Goblins, which include a 5.5 inch TFT LCD display with 2,560 × 1,440 resolution and a refresh rate of 90 Hz. A Qualcomm Snapdragon 820 CPU is built into the VR headset, which allows for wireless delivery of VR. The headset weighed 500 g (about 2 lbs). Head movement was used to navigate through the virtual environment and participants used the lateral button on the headset to make selection within the virtual scenes.

Participants in the AO-GI group received NIUTA wired in-ear earbuds with a 3.5 mm audio jack, which was used to connect to and play audio from a tablet.

All participants received a 8-inch Lenovo tablet to make their pre and post GI pain ratings. AO-GI group participants also used the tablet to play the auditory-only guided imagery experiences. The tablet had a quad-core processor, 1.4 GHz, and 16GB storage.

### VR-GI and AO-GI experiences

**Figure 1:**
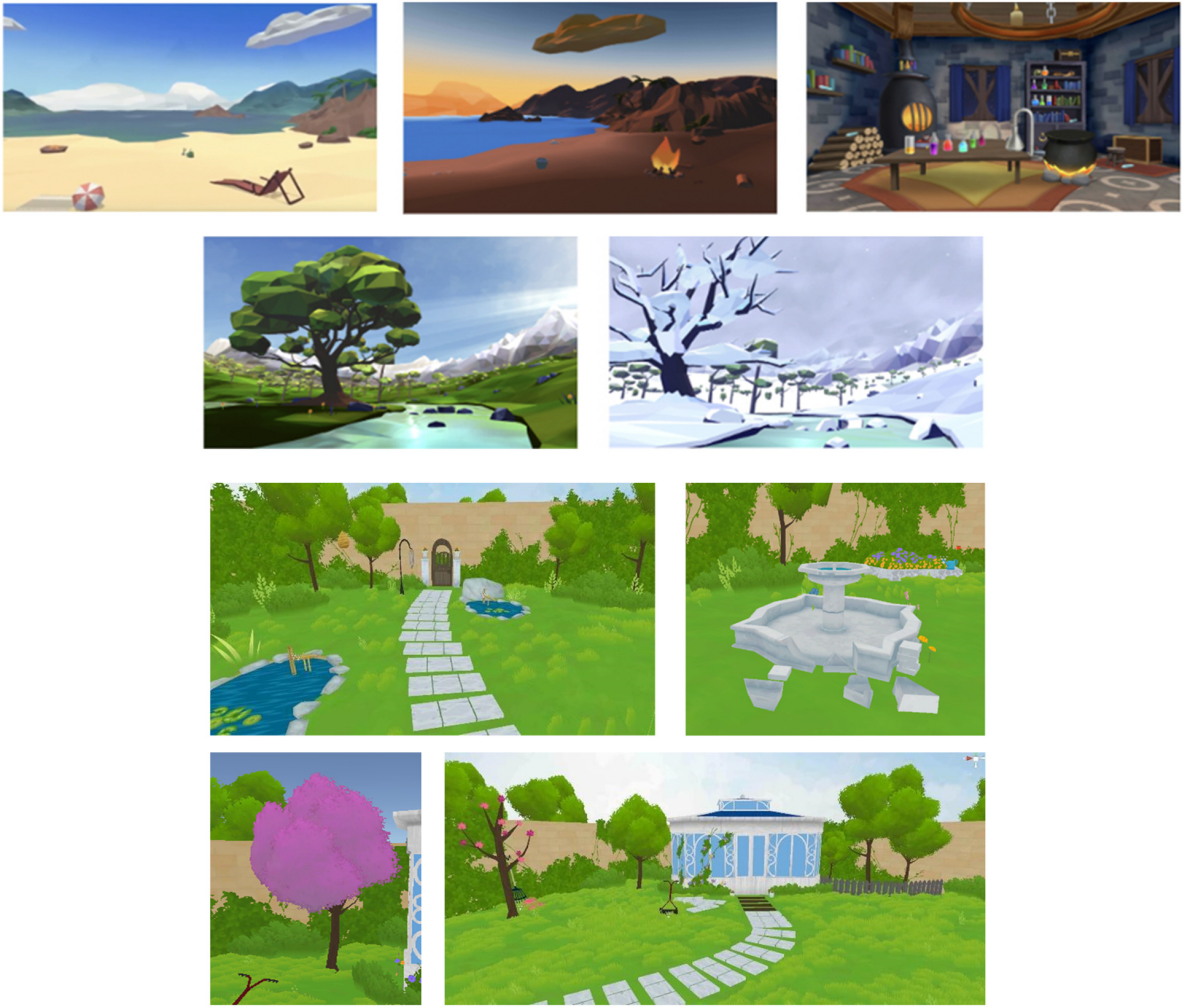
The garden and choose your own adventure VR-GI experiences. Images used with permission of Eran Orr, CEO, XRHealth Inc.

The VR-GI and AO-GI proprietary programs (BehaVR Inc., dba RealizedCare; Elizabethtown, KY; https://www.realizedcare.com) were designed and developed by psychologists from USC Pain Center in collaboration with Limbix Health Inc. (San Francisco, CA), a for-profit company that was operational during the trial period but is no longer in operation. The VR-GI program was delivered using a consumer grade PICO Goblin VR head-mounted display (PICO Interactive, San Francisco, CA) and offered two computer-generated experiences: The Garden and Choose Your Own Adventure (Figure 1). Each experience had a duration of approximately 15 min, the suggested optimal time for continuous VR use (39). The AO-GI audio tracks had the same narration, without reference to visual elements, and overall duration to the VR-GI experience. The duration of the GI experiences was also consistent with previous studies evaluating AO-GI (3), with brief AO-GI exercises having been observed to be effective in reducing chronic pain in as little as 1–2 min (40).

The Choose Your Own Adventure experience began with participants creating a pain object and, assigning it a visual identity (e.g., shape, color, and texture) to externalize their discomfort. Next, participants had the opportunity to explore 5 different environments with various locations: a castle chamber, a spring meadow, a beach at daylight, a beach at sunset, and a winter landscape. Participants reached these different environments through an elevator accompanied by a guided relaxation exercise, with a focus on progressive muscle relaxation. The experience incorporated structured grounding tasks based on sensory exploration techniques, revolving around sight, sound, and touch to help regulate anxiety and panic symptoms. Participants engaged in interactive activities that encouraged mindfulness and environmental awareness through sight exploration (e.g., uncovering items beneath sand or snow, observing light and atmospheric changes, or mixing magical potions in the castle), sound exploration (e.g., actively listening to environmental sounds such as waves, crackling fire, wind, or a babbling brook), and touch exploration (e.g., using visualization to simulate tactile engagement, such as feeling the texture of seashells, the weight of tree branches, the warmth of fire, or the sensation of lifting clouds from the sky). The narration guided participants through paced breathing exercises and relaxation cues, gradually deepening their engagement with the chosen environment. The interactive elements allowed participants to actively shape their surroundings, reinforcing a sense of control and presence in the moment. The session concluded with a reflection exercise, where participants revisited their “pain object”—a visual representation of their discomfort—and modified its characteristics (e.g., changing its size, shape, or texture) based on how they would like their pain to look and feel. This final step reinforced the idea that pain perception can be modulated through cognitive reframing and sensory engagement.

The Garden experience, where patients engaged in virtual tasks to restore a garden, was designed around sensory immersion and engagement qualities to increase self-efficacy in pain coping and to mitigate pain via gate control mechanisms, which centers on the idea that non-pain related types of stimuli can reduce pain perception. As in the Choose Your Own Adventure experience, participants first created a pain object, assigning it a visual identity (e.g., shape, color, and texture) to externalize their discomfort. However, this object soon caused disruptions within a virtual garden, symbolizing the uncontrolled nature of pain. To restore balance, participants engaged in structured virtual tasks that mimicked mindful actions associated with pain modulation, such as: closing garden gates along their path, reinforcing the idea of controlling pain pathways and reducing its free movement in their environment; Engaging in activities such as repairing a fountain, adding color to faded areas, or trimming overgrown foliage, symbolizing actively restoring balance and control over pain; and selecting natural sounds (e.g., wind in trees, running water) to reinforce attentional redirection away from painful stimuli, a core principle of gate control mechanisms. As with Choose Your Own Adventure, participants were guided by paced breathing exercises and mindfulness cues, helping to cultivate relaxation and cognitive reframing. The experience culminated in a self-reflection phase, where participants revisited their pain object and adjusted its characteristics according to how they would like their pain to look and feel, symbolizing the potential to regulate pain perception and reinforcing a sense of control over their pain to increase self-efficacy.

The mechanisms designed to impact pain perception were consistent between the VR-GI and AO-GI group, as the AO-GI audio tracks had nearly identical narrations to the VR-GI experiences. The only difference between VR-GI and AO-GI experiences was that, whereas participants in VR-GI were able to see, hear, and directly interact with elements of the virtual environment, the AO-GI narration prompted participants to *imagine* seeing, hearing, and directly interacting with the environments in the same way.

## Measures

### Primary outcomes

#### Study & intervention feasibility

Metrics to determine feasibility were (a) uptake rate (proportion of eligible participants who chose to enroll in the study out of the total number of eligible participants), (b) study tolerability (proportion of enrolled participants who discontinued intervention or were lost to follow-up out of the total number of enrolled participants), and (c) adherence rates (the average percentage of daily VR sessions completed by each participant over the 14 days intervention period). Session completion was measured using data and time stamps extracted from the VR headsets and was defined as spending at least 15 min on the headset daily, which was the minimum duration of a VR experience.

#### Acceptability & patient experience

##### Self-report questionnaire

To better understand participants’ experiences with the intervention, a 23-item self-report questionnaire was administered post-intervention to the VR-GI group only. The questionnaire was developed by the study authors and was not a validated measure but was designed to capture qualitative and quantitative insights into participants' perceptions and experiences of VR-GI. It included questions related to willingness to recommend VR-GI to a friend, impact of the intervention on mood, pain, control over pain, use of painkillers, comfort of the VR headset, ease of use, ease of integrating VR into daily routine, and enjoyment and perceived utility of each of the two VR experiences. These questions were rated on Likert scales from 0 to 10. Participants were also asked to indicate which VR experience they preferred more and why. Additionally, the survey included free response questions that gave participants the opportunity to report any adverse effects of the VR-GI, to explain what they liked or disliked about either of the two VR experiences, to indicate what, if anything, they would change about either VR experience, and to share any other general comments about the VR-GI intervention. Three additional survey questions captured information for the product developers unrelated to the study goals and, therefore, are not reported here.

**Table 2:** Self-report questionnaire.

Question (all questions on a 0–10 scale 0–10, where 0 = not at all and 10 = completely)	Mean	SD
How willing would you be to recommend Limbix Virtual Reality Guided Imagery to a friend?	7.05	3.07
How much did Limbix Virtual Reality Guided Imagery practice help improve your mood?	6.76	2.34
How much did Limbix Virtual Reality Guided Imagery practice help you manage your pain?	5.57	2.33
How much did Limbix Virtual Reality Guided Imagery practice make you feel more control over your pain?	5.90	2.38
How much did Limbix Virtual Reality Guided Imagery practice help you reduce your use of painkillers?	2.58	3.39
How willing would you be to continue using Limbix Virtual Reality Guided Imagery to manage your pain?	7.00	3.51
How comfortable did you find wearing the Virtual Reality Headset?	4.71	3.23
How easy was the Limbix Virtual Reality hardware to use?	8.76	2.41
How easy was it to integrate Limbix Virtual Reality Guided Imagery into your daily routine?	8.38	1.91
How much did you enjoy Virtual Reality Experience #1 (The Garden)?	6.29	2.53
How useful did you find Virtual Reality Experience #1 (The Garden)?	6.48	2.46
How much did you enjoy Virtual Reality Experience #2 (Choose your own adventure)?	7.25	2.65
How useful did you find Virtual Reality Experience #2 (Choose your own adventure)?	6.50	2.65

#### Secondary outcomes

To conduct a preliminary evaluation of the efficacy of the interventions several pain-relevant outcome measures were administered to participants at baseline and post-intervention.

#### Pain intensity

The Pain Numeric Rating Scale was used to assess each participant's self-reported pain intensity on a scale of 0–10, with higher scores indicating higher self-reported pain (41): Patients were asked to indicate the intensity of their pain levels on a scale of 0–10 (0 = no pain, 1–3 = mild, 4–6 = moderate, 7–9 = severe, 10 = worst pain imaginable). They were asked to do this rating for their lowest, highest and average pain scores during the last 7 days. Pain intensity ratings were collected at baseline and post-intervention. Clinical significance of pain reduction is considered to be 30% (42).

#### Anxiety

The Generalized Anxiety Disorder Assessment (GAD-2) is a 2-item self-reported assessment that screens for anxiety disorders. The total score ranges from 0 to 6 with higher numbers indicating worse anxiety outcomes (43). Clinical significance was defined as a 20% reduction in anxiety symptoms (44).

#### Depression

The Patient Health Questionnaire (PHQ-2) is a 2-item self-reported assessment for depression. The total score ranges from 0 to 6 with higher numbers indicating worse depression outcomes (45, 46). Clinical significance was defined as a 20% reduction in depression symptoms (44).

#### Physical and mental health

The Short Form Health Survey (SF-12) is a 12-item self-reported assessment for health-related quality of life measured by two composite scores (physical and mental health scores). The physical and mental health scores both range from 0 to 100 with higher numbers indicating better health outcomes (47). Minimally clinical importance difference for both subscales of the SF-12 was defined as a mean change of 5 points (48).

### Data analysis strategy

We first present basic demographic characteristics of the study sample followed by descriptive and qualitative results related to feasibility, acceptability, and patient experience. For the self-report questionnaire, means and standard deviations of responses on the Likert scale questions were calculated as well as a count of participants' preference between the two VRGI experiences. A grounded theory approach was used to analyze free response answers on the self-report questionnaire to derive emergent themes (49). Themes were generated through an inductive process of coding involving iteratively categorizing and sorting data through pattern recognition (50, 51). Notes were reviewed, iteratively coded, and grouped into themes until consensus on key themes was reached among study authors. Sample quotes by participants are included for illustrative purposes. For the preliminary efficacy testing, we present the means, standard deviations, and the percent difference between baseline and post-intervention within each of the two groups for each of the constructs. Given that the primary focus of the present study was evaluating feasibility, the study was not powered for significance testing, and thus between-group and within-group significance testing was not conducted.

## Results

### Demographics

Participant demographics are presented in Table 1.

**Table 1:** Baseline demographic characteristics by group and total.

Demographics	VR-GI (*n* = 24)	AO-GI (*n* = 12)	Total
Gender, *n* (%)			
Male	8 (33.3%)	2 (16.7%)	10 (27.7%)
Female	16 (66.7%)	10 (83.3%)	26 (72.2%)
Ethnicity, *n* (%)			
Hispanic/Latino	4 (16.7%)	1 (8.3%)	5 (13.9%)
Race, *n* (%)			
White	18 (75%)	9 (75%)	27 (75%)
American Indian or Alaska Native	2 (8.3%)	0	2 (5.6%)
Asian	0	0	0
Native Hawaiian or Other Pacific Islander	0	0	0
Black or African American	0	1 (8.3%)	1 (2.7%)
More than one race	0	1 (8.3%)	1 (2.7%)
Unknown or not reported	4 (16.7%)	1 (8.3%)	5 (13.8%)
Age, mean (SD)			
Age, mean (SD)	54.9 (13.1)	56.1 (11.5)	54.9 (12.8)

### Study and intervention feasibility

96 participants were screened for eligibility. 15 (15.6%) did not meet enrollment criteria (pain less than 5/10 or neck pain/ neck movement problems), 15 (15.6%) declined to participate, and 30 (31.3%) were unable to participate for other reasons, including failure to schedule a consent session and family circumstances. Ultimately, 36 participants (*n* = 24 randomized to the VR-GI group and *n* = 12 randomized to the AO-GI group) were enrolled in the study resulting in a 44.4% uptake rate (36 out of 81 eligible participants). Study tolerability, as measured by trial completion rate, was 88.9% (32 out of 36 participants), with one participant in the VR-GI group discontinuing the intervention due to difficulty using the VR headset and three participants (2 VR-GI group, 1 AO-GI group) lost to follow-up.

Of the 21 participants in the VR-GI group who completed the study, the adherence rate was 66.4%. The average number of completed VR sessions was 9.3 over the 14-day intervention period.

### Acceptability and patient experience

Means and standard deviations for all Likert scale questions on the self-report questionnaire are reported in Table 2.

#### Acceptability

In terms of acceptability, VR-GI participants gave high ratings for ease of integrating the VR intervention into their daily routine (mean = 8.38, SD = 1.91) and for ease of using equipment in the self-report questionnaire (mean = 8.76, SD = 2.41). On the other hand, the average rating for equipment comfortability was moderately low with high variability across the sample (mean = 4.71, SD = 3.23). Of the 21 VR-GI participants who completed the study, 5 participants (23.8%) reported non-serious adverse events such as dizziness, nausea, and headaches.

In free response feedback, a few participants reported that the headset was uncomfortable, heavy, and required excessive mobility which could trigger neck pain, pressure on the nose, and headaches. Four examples follow:

“… the ideal scenario would have been in a reclined position only requiring 120 degrees of head movement from side to side. This would have allowed the participant to be in a relaxed state and not in any positions that triggered a flare to their CRPS. Additionally, having to touch the headset button to click, that should be replaced with a handheld ‘clicker’ that could be operated by the other hand and not require the participant to repeatedly reach up and touch the visor.” (Male, Age: 57)

“… the weight of the headset irritated my neck somewhat. Over time, I became used to the weight, and it was not really a problem by the end of the study. However, I would not be able to wear the headset for an extended time…” (Female, Age: 62)

“… the nose piece was uncomfortable; I would prefer that it was a soft material like around the eyes.” (Female, Age 37)

“… difficult to focus as unable to wear my prescription glasses.” (Female, Age 58)

Participants also commented on the design and mechanics of the intervention. For instance, two participants remarked that the sound was too low even at maximum volume. Others did not like the narrator's voice or the graphics, which they felt were not very realistic. Three quotes follow:

“Overall, I really enjoyed the voice of the narrator and the pace and style of his spoken guidance. That voice relaxed me and over time it grew familiar and worked faster to block out the world. However, the limits of the image quality in VR bothered me…” (Female, Age: 62)

“Maybe the graphics could have been improved upon a little bit too. Made more realistic…” (Female, Age 64)

“… the recording was done at very low volume.” (Male, Age 57)

Finally, in terms of the content of the imagery, many felt as if some scenarios could be triggering their chronic pain conditions. A quote follows:

“Too often, the wording overly focuses on touch sensation that could trigger a pain flare-up for CRPS patients. Cold floors, sand, snow, the list goes on and on. Rather than going for extremes of summer and winter, I would have picked something more neutral like a green meadow or forest setting, I would have used more words to focus on smell, hearing, visual components, or possibly how things feel on the face rather than the hands or feet.” (Male, Age: 57)

#### Patient experience

Overall, participants in the VR-GI group reported moderately high enjoyment of the VR experiences (The Garden: mean = 6.29, SD = 2.53; Choose Your Own Adventure: mean = 7.25, SD = 2.65) and indicated they would be willing to continue using the VR-GI experiences to manage their pain, though variability was high (mean = 7.0, SD = 3.51). Free response feedback suggested that enjoyment of the VR experiences and willingness to continue using VR-GI may, in part, have been related to the “game-like” quality of the VR experiences, which some participants reported helped to take their minds off their pain, and to the ability of VR to engage participants’ senses and impact pain coping and mental wellbeing.

Five quotes follow:

“I was surprised how much I like the castle location—I'm not a potions or crystals person, but it was kind of fun and so different from all the other locations that I was able to lose myself more easily” (Female, Age 62)

“VR used more of my senses keeping me more engaged” (Female, Age 38)

“I loved the experience of designing my pain and what it looked like to me. Being able to visualize the pain I feel constantly made me feel as though I had more control over it. I had a really bad flare up during this trial, and although the guided imagery didn't decrease my pain by a lot, it was still very helpful in coping with it.” (Female, Age 21)

“… I felt more improvement from a mental health perspective…” (Female, Age 37)

“I would like to know if/when something like this is available for purchase. I felt a benefit from the VR as I was able to mentally escape. I would use this as a tool in my pain management.” (Female, Age 37)

Interestingly, most participants (*n* = 15 out of 21) reported preferring the Choose Your Own Adventure experience over The Garden experience. (Only four participants reported preferring The Garden experience; two participants indicated no preference.) Free responses suggested that this preference was driven by greater customization and choice built into the Choose Your Own Adventure experience.

### Preliminary efficacy

Table 3 shows the baseline and post-intervention means and standard deviations for each outcome by group.

**Table 3:** Within-group changes in outcomes by group.

Outcome measure	VR-GI		AO-GI	
	Baseline	Post	Baseline	Post
Pain numeric rating scale				
Lowest pain, mean (SD)	4.00 (1.82)	3.71 (1.95)	3.92 (1.51)	3.64 (1.63)
Highest pain, mean (SD)	8.42 (0.83)	7.86 (1.01)	7.75 (1.36)	7.40 (0.97)
Average pain, mean (SD)	6.08 (1.18)	5.62 (1.50)	5.75 (1.42)	5.45 (1.44)
GAD-2				
Anxiety, mean (SD)	1.83 (1.86)	0.90 (1.17)	1.58 (1.68)	1.20 (1.55)
PHQ-2				
Depression, mean (SD)	2.00 (1.53)	0.86 (1.39)	1.33 (1.44)	1.30 (1.70)
SF-12				
Mental health, mean (SD)	43.61 (12.31)	50.43 (10.18)	43.45 (13.08)	48.55 (16.24)
Physical health, mean (SD)	27.02 (6.87)	27.93 (8.61)	29.06 (4.80)	27.74 (7.22)

From baseline to post-intervention, pain ratings for the VR-GI group all showed a greater numerical decrease in pain (NRS) compared to pain ratings for the AO-GI group. The VR-GI lowest pain score decreased by 8.74% (baseline lowest pain score = 4.0, post-intervention lowest pain score = 3.71), whereas the AO-GI lowest pain score decreased by 5.91% (baseline lowest pain score = 3.92, post-intervention lowest pain score = 3.64). The VR-GI group highest pain score decreased 6.96% (baseline highest pain score = 8.42, post-intervention highest pain score = 7.86) compared to a 1.18% increase in the AO-GI group (baseline highest pain score = 7.75, post-intervention highest pain score = 7.4). The VR-GI group average pain score decreased 8.22% (baseline average pain score = 6.96, post-intervention average pain score = 6.08) compared to a 2.52% decrease in the AO-GI group (baseline average pain score = 5.75, post-intervention average pain score =  5.45).

From baseline to post-intervention, the VR-GI group showed a greater numerical decrease in anxiety compared to the AO-GI group. The within-group reduction in anxiety was 43.9% (baseline GAD-2 score = 1.83, post-intervention GAD-2 score = 1.0) in the VR-GI group. In the AO-GI group, the reduction was 22.3% (baseline GAD-2 score = 1.58, post-intervention GAD-2 score = 1.2).

The VR-GI group also showed a greater numerical decrease in depression compared to the AO-GI group from baseline to post-intervention. The within-group reduction in depression was 52.3% (baseline PHQ-2 score = 2.0, post-intervention PHQ-2 score = 0.86) in the VR-GI group. In the AO-GI group, the reduction was 5% (baseline PHQ-2 score = 1.33, post-intervention PHQ-2 score = 1.30).

From baseline to post-intervention for the SF-12 Mental Component score, the VR-GI group showed a greater numerical improvement in mental health compared to the AO-GI group. The within-group increase in mental health was 21.6% (baseline mental health score = 43.61, post-intervention mental health score = 50.43) in the VR-GI group. In the AO-GI group, the increase was 13% (baseline mental health score = 43.45, post-intervention mental health score = 48.55).

From baseline to post-intervention for the SF-12 Physical Component score, the VR-GI group showed a 3% increase in physical health (baseline physical health score = 27.02, post-intervention physical health score = 27.93). The AO-GI group showed a 5% decrease in physical health (baseline physical health score = 29.06, post-intervention physical health score = 27.74).

## Discussion

The aim of this study was to evaluate the feasibility, acceptability, patient experience, and preliminary efficacy of an at-home VR-GI intervention for patients with chronic pain. The results collectively indicate the study and intervention were feasible as evidenced by the high retention rate and high study adherence (27, 28, 31, 52, 54). Participant feedback suggested some acceptability concerns in terms of the hardware and software, though there was a low incidence of adverse events. Overall, patient experience was positive and the intervention demonstrated promising preliminary efficacy based on improvements in pain, depressive symptoms, anxiety symptoms, and quality of life.

### Feasibility and self-report responses

Retention and study uptake rates, respectively 88.9% and 44.4%, were relatively high (28, 52, 54). Recruitment through physician referrals are a possible factor driving high uptake rates. At-home interventions coupled with video calls allowed for greater flexibility during the trial, which may have contributed to high recruitment and retention. Additionally, assessments were conducted solely online with assessment reminders from research coordinators, which could have contributed to the high retention rate.

Study adherence at 66.4% when looking at VR headset data (completion of at least one 15-min session per day) was also notably high in comparison to previous at-home VR studies, which have had low and highly variable adherence, with a meta-analysis study on VR for pain treatment defining strong adherence to be 64% (52, 53). High study adherence could have also been influenced by reminders from research coordinators as well as the low recommended frequency and duration of intervention, which was daily and at least for 15 min. Future studies could be conducted over longer trial periods with less participant support from research coordinators to further assess overall adherence in a more naturalistic context.

In terms of acceptability, participant feedback was mixed. VR-GI had expected side effects of dizziness and nausea; these are well-known and common side effects to VR (55). These adverse effects, in addition to feedback around the weight of the headset and the impact of the headset and VR-GI experiences in triggering pain, could have contributed to the moderately low ratings of comfortability in participant feedback. This feedback may have been driven by participants who had chronic neck or hand pain who reported discomfort with the amount of movement that the VR experiences required. Though there was noted discomfort associated with VR-GI, high ratings of ease of integration and use of the VR hardware, do support the acceptability of the intervention. It is important to note that the average age of participants was 56.4 years and most reported having never previously experienced VR, thus the relatively high ratings for ease of use and ability to fit VR-GI into a daily routine are encouraging. Future studies should consider excluding individuals with neck or hand pain and/or consider using a handheld remote or lighter headset. Participant feedback around changes to the content of the VR-GI experiences to reduce pain triggering content can also be incorporated into subsequent versions of the VR-GI experiences.

Feedback around participant experience was largely positive. Though participants reported that VR-GI did not specifically reduce their pain, VR was rated as a helpful coping mechanism in managing their pain. Patients reported that they would be likely to recommend VR-GI to a friend and they wished to continue using it.

### Preliminary efficacy

Though the study's small sample size limits conclusions around efficacy, average and highest pain scores showed numerically greater reductions in the VR-GI group compared to the AO-GI group. However, these effects were well below a clinical significance threshold. On the other hand, mental health outcomes as measured by the GAD-2, PHQ-2, and SF-12 mental health subscale all demonstrated numerical improvements in mental health outcomes that met clinical significance thresholds. Participant qualitative feedback also supported improvements around mental health. This preliminary evidence points to a potential impact of VR-GI in particular on mental health outcomes, in line with theoretical perspectives that VR-GI reduces pain via top-down modulation of pain pathways by cognitive and emotional brain regions. Future research is necessary to examine the efficacy and clinical significance of VR-GI to improve chronic pain and mental health outcomes via a larger, statistically powered clinical trial, to assess potential for long-term efficacy and symptom remission with longer term follow-up timepoints, and to further explore the mechanisms by which VR may impact chronic pain.

### Limitations

Although recruitment was aimed at participants with back pain and CRPS, neck pain was not specifically listed as an exclusion criterion and may have contributed to complications in using VR for participants who also had neck pain.

Future iterations of the VR headset can improve the logging system to more accurately monitor engagement metrics. Additionally, VR as a delivery method for pain management has logistical barriers such as discomfort from weight of hardware and equipment and lack of accessibility due to price.

## Conclusions

This study demonstrated the feasibility, acceptability, and preliminary efficacy of multiple sessions of at-home VR-GI as a promising, non-pharmacological treatment for chronic pain. Findings indicate that using VR across consecutive days is feasible as a treatment that can be independently administered by patients with chronic pain at home. Nevertheless, results need to be replicated and future studies should investigate long-term efficacy, adherence and engagement to further validate these findings. Additionally, future iterations of the intervention should focus on improving VR logging capabilities and the comfortability of the VR equipment.

## Data Availability

The datasets presented in this article are not readily available because this research is a clinical trial conducted under FDA regulations. Direct identifiers and/or the key to the codes will be destroyed as directed by the sponsor (IND/IDE holder) in accordance with FDA regulations. The NIH requires that the records be retained for three years following the completion of the study. Requests to access the datasets should be directed to faye.weinstein@med.usc.edu.
